# Impacts of the novel coronavirus SARS-CoV-2 on wildlife behaviour via human activities

**DOI:** 10.1371/journal.pone.0285893

**Published:** 2023-05-16

**Authors:** Haruka Uehara, Wakana Nishiyama, Shirow Tatsuzawa, Keiji Wada, Takashi Y. Ida, Yoichi Yusa

**Affiliations:** 1 Faculty of Science, Nara Women’s University, Nara, Japan; 2 Graduate School of Humanities and Human Sciences, Hokkaido University, Sapporo, Japan; Centre de Recherche Scientifique et Technique sur les Regions Arides, ALGERIA

## Abstract

Severe acute respiratory syndrome coronavirus 2 (SARS-CoV-2) caused the pandemic of the coronavirus disease 2019 (COVID-19), resulting in a global lockdown in 2020. This stagnation in human activities (‘anthropause’) has been reported to affect the behaviour of wildlife in various ways. The sika deer *Cervus nippon* in Nara Park, central Japan, has had a unique relationship with humans, especially tourists, in which the deer bow to receive food and sometimes attack if they do not receive it. We investigated how a decrease and subsequent increase in the number of tourists visiting Nara Park affects the number of deer observed in the park and their behaviour (bows and attacks against humans). Compared with the pre-pandemic years, the number of deer in the study site decreased from an average of 167 deer in 2019 to 65 (39%) in 2020 during the pandemic period. Likewise, the number of deer bows decreased from 10.2 per deer in 2016–2017 to 6.4 (62%) in 2020–2021, whereas the proportion of deer showing aggressive behaviour did not change significantly. Moreover, the monthly numbers of deer and their bows both corresponded with the fluctuation in the number of tourists during the pandemic period of 2020 and 2021, whereas the number of attacks did not. Thus, the anthropause caused by the coronavirus altered the habitat use and behaviour of deer that have continuous interactions with humans.

## Introduction

Severe acute respiratory syndrome coronavirus 2 (SARS-CoV-2) caused the coronavirus disease 2019 (COVID-19) pandemic [1–3], via zoonotic transmission [4]. By December 2022, the total number of infected people exceeded 600 million [5]. Due to the pandemic, there was a stagnation in human activities (termed ‘anthropause’), including tourism, around the world starting in 2020 [6, 7]. Such interruptions in human activities provide an opportunity to shed light on the interactions between humans and various wildlife that have been otherwise masked, by observing their responses to the anthropause [8–12]. Understanding the relationships between humans and wildlife is crucial because increasing human populations are rapidly changing the global environment and thus have fundamentally affected many wild animals [8, 13–15].

As examples of the effects of anthropause on wildlife behaviour in 2020–2021, a change in the singing performance of the white-crowned sparrow *Zonotrichia leucophrys* was reported before and during the San Francisco urban lockdown [16]. Additionally, the eastern cottontail rabbit *Sylvilagus floridanus* in Italy was significantly more active during the day in 2020 than in pre-pandemic years [17]. Concerning impacts on wildlife populations, 80% of 82 bird species in Canada and the United States increased their population sizes during the pandemic period (2019–2020) compared with the pre-pandemic period (2017–2018) in urban areas [18]. These examples indicate that reduced human activity has weakened human effects on wild animals [19]. However, currently, many animals have become adapted to urban or other human-mediated habitats. Some animals have even learned complicated behaviour through continuous interactions with humans. For instance, tits in England have learned to open the lids of milk bottles [20], and bottlenose dolphins in southern Brazil have cooperated with net-casting fishers to catch the same prey (the mullets) [21]. However, the responses to the anthropause of animals under strong human influence have been little studied [22].

**Fig 1:**
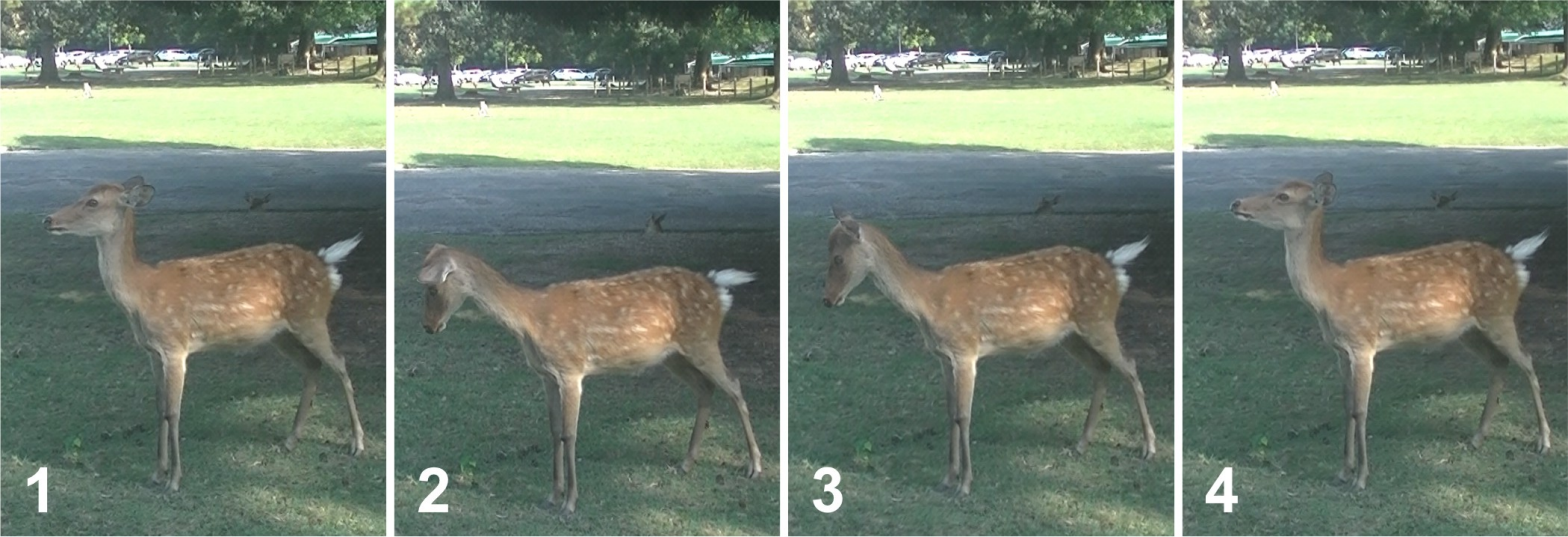
Bowing behaviour displayed by sika deer in Nara Park. 1) Facing forwards (towards the direction of the feeder) at the beginning, 2) ears forwards and head down, 3) head up again, 4) facing forwards again.

Nara Park (6.6 km^2^) in Nara City, central Japan, is visited every year by many tourists from all over Japan and abroad. Nara Park, including adjacent areas such as Kasugayama Primeval Forest, has a population of 1,050–1,400 wild sika deer *Cervus nippon*, according to the Nara Deer Foundation (https://naradeer.com). The sika deer here are often fed ‘deer cookies’ sold in the park or other artificial food items. The density of deer occurrence is higher in tourist spots in Nara Park than in adjacent areas, including Kasugayama Primeval Forest, although deer migrate between these areas [23]. Moreover, the sika deer display characteristic “bowing" behaviour that resembles bowing to human feeders (Fig 1, S1 Video). Such bowing behaviour is almost exclusively observed in Nara Park and is considered to have developed through interactions with humans for many years [24]. Conversely, aggressive behaviour is sometimes observed when deer do not receive cookies from humans.

In 2020 and 2021, the number of tourists visiting Nara Park greatly decreased due to governmental restrictions on travel and other activities to control the spread of COVID-19. During the first phase of the emergency declaration (from 16 April to 14 May 2020), the number of visitors to Nara Park decreased by 84.8% compared to the previous year [25]. This anthropause reduced opportunities for human–deer interactions. Thus, the pattern of habitat use and behaviour of the deer may have changed in response to the anthropause.

Because deer in Nara Park regularly obtain artificial food as a reward from humans, we hypothesised that the anthropause caused by COVID-19 i) reduced the number of deer utilising Nara Park. As the rewards from bowing and attacks decrease during the pandemic period, we considered that ii) the number of bows and attacks on feeders per feeding event would also decline. However, as deer have memories of receiving foods from humans, iii) such a decrease would be temporary, and the site use and behaviour of deer recover with the gradual increase in visitors to Nara Park. To test these predictions, we conducted field surveys on the number of deer observed, the number of deer bows and displays of aggressive behaviour towards a feeder in Nara Park. Our study aimed to investigate the impact of changes in tourist numbers in Nara Park caused by COVID-19 on the behaviour of sika deer by surveying monthly from June 2020 to June 2021 and comparing the results with the corresponding data from 2015 to 2019.

## Methods

### Survey site and period

From 2015 to 2021, surveys were conducted on sika deer (*Cervus nippon*) at three sites in Nara Park: the area around Todai-ji Temple Nandai Gate (Todai-ji site: 34°41’07” N; 135°50’23” E, 1.8 ha), the area around Nara National Museum (Museum site: 34°41’01” N; 135°50’10” E, 4.2 ha), and the area around Ukigumo and Kasugano Park (Ukigumo site: 34°41’03” N; 135°50’30” E, 6.4 ha). All the three sites were defined by clear borders, such as streams, pavements, trees, and lawns. All surveys were conducted on weekdays. These sites are constantly visited by tourists because of the famous Great Buddha Statue and other national treasures. Deer cookies are sold in shops at these sites, and deer have the chance to obtain deer cookies from tourists.

### Number of deer in study sites

We studied the number of deer once in April during 2015–2019 (i.e., before the COVID-19 pandemic) and monthly from June 2020 to June 2021 (i.e., during the pandemic). The months surveyed at the sites were April 2015–2019, June–November 2020, and March–June 2021 at the Todai-ji site; June–December 2020 and January–June 2021 at the Museum site; and June–November 2020 and June 2021 at the Ukigumo site. Unfortunately, we could not conduct the surveys from April to May 2020 because the Japanese government declared emergency statement. We carried out a route census at each site three times a day (13:00, 14:00, and 15:00) and counted the number of deer (separately for fawns, males, and females). Fawns were defined as individuals who were considered to have been born within 1 year, judging from their small size, white spots, and facial features [26]. Males and females were defined as larger individuals with and without antlers, respectively. Each site was small enough to walk around and count all the deer without double counting. In addition, we counted the number of people during the route census from June 2020 to June 2021 in the same way as the number of deer.

### Responses of deer to human feeders

To compare the behaviours of deer to human feeding before and during the COVID-19 pandemic, we recorded deer reactions at the Museum site during September 2016–January 2017 and June 2020–June 2021 from 15:00 to 16:00 using a video camera (GZ-R 480; JVC Kenwood, Yokohama). We randomly selected approximately 20 deer (both males and females) per month. Fawns were not included in this study because they had not yet learned bowing behaviour or were in the process of learning [24]. We stood 1 m away from a deer and showed it cookies but without giving the cookies to the deer. We recorded the number of bows per deer in response to the presentation of deer cookies until the deer either attacked the feeder (by either biting, kicking, or head-butting) or gave up and moved away. A bow is defined as a series of downwards and upwards movements of the head pointed towards the feeder. Data were collected only once from the same individuals in each month. Although we could not totally rule out the possibility of double counting over time, such possible double counting would not be large as there have been over 1,000 deer living in Nara Park.

### Data analysis

All analyses were conducted using either generalised linear models (GLMs) or generalised linear mixed models (GLMMs) as implemented with the GLIMMIX procedure of SAS version 9.4 (SAS Institute Inc., Cary, NC, USA). All analyses of count data (i.e., numbers of deer and deer bows) involved a negative binomial distribution and ln-link function.

The effect of tourists on the number of deer was analysed with both GLM and GLMM. First, the effect of the annual number of tourists on the number of deer at the Todai-ji site was analysed using GLM with deer data collected in April 2015–2019, June 2020, and April and June 2021. Unfortunately, no data were available on the number of visitors to Nara Park before 2020; thus, the annual number of tourists visiting Nara City was obtained from the website of Nara City (https://www.city.nara.lg.jp) and used for the analysis. In the statistical model, the logarithm of the number of tourists was incorporated as a covariate, and month (April or June) was incorporated as a fixed factor. Next, the number of deer in the monthly survey (June 2020–June 2021) at the three sites was analysed with the number of tourists counted simultaneously as a covariate. To account for differences in the area of the three sites, the number of deer per site area (logarithm of site area in ha) was included as an offset variable. A mixed model was necessary for this analysis. Since the surveys were conducted on a monthly basis, errors were assumed for each month, so we used GLMM with a model of variance components to account for the correlated responses associated with block design. In all cases, including the GLMM below, the method of Kenward & Roger (1997) was used to adjust the (possibly fractional) denominator degrees of freedom for the *F* tests to account for the estimated correlated responses [27].

The number of deer bows and the display of deer aggression were analysed with GLMMs in two ways. First, analyses of the number of deer bows and deer aggression for yearly data (pre-pandemic years of 2016–2017 or pandemic years of 2020–2021) assessed the effects of year, month, and their interaction as fixed factors. The number of bows and the presence/absence of aggression by the deer were analysed using negative binomial and binomial distributions and ln-link and logit link functions, respectively. Since both male and female deer were observed, errors were assumed for each sex, so we used GLMM with a model of variance components to account for the correlated responses associated with this block design. Second, analyses of the number of bows and display of aggression by deer for monthly data (June 2020–June 2021) assessed the effects of the logarithm of the number of tourists as a covariate. In the former, a negative binomial distribution and ln-link function were involved, and in the latter, a binomial distribution and logit link function were involved. For the same reason, these models also included sex (male and female) as a random factor.

To facilitate interpretation, we present results for a particular factor or covariate adjusted for the effects of other components of statistical models. For categorical factors, we present least-squares means and their standard errors [28]. Specifically, we used the estimated linear model to predict the dependent variable for each observation, given the observed value of the independent variable of interest and the means of all other independent variables, to which we added the observation’s residual.

## Results

### Numbers of deer and tourists

**Fig 2:**
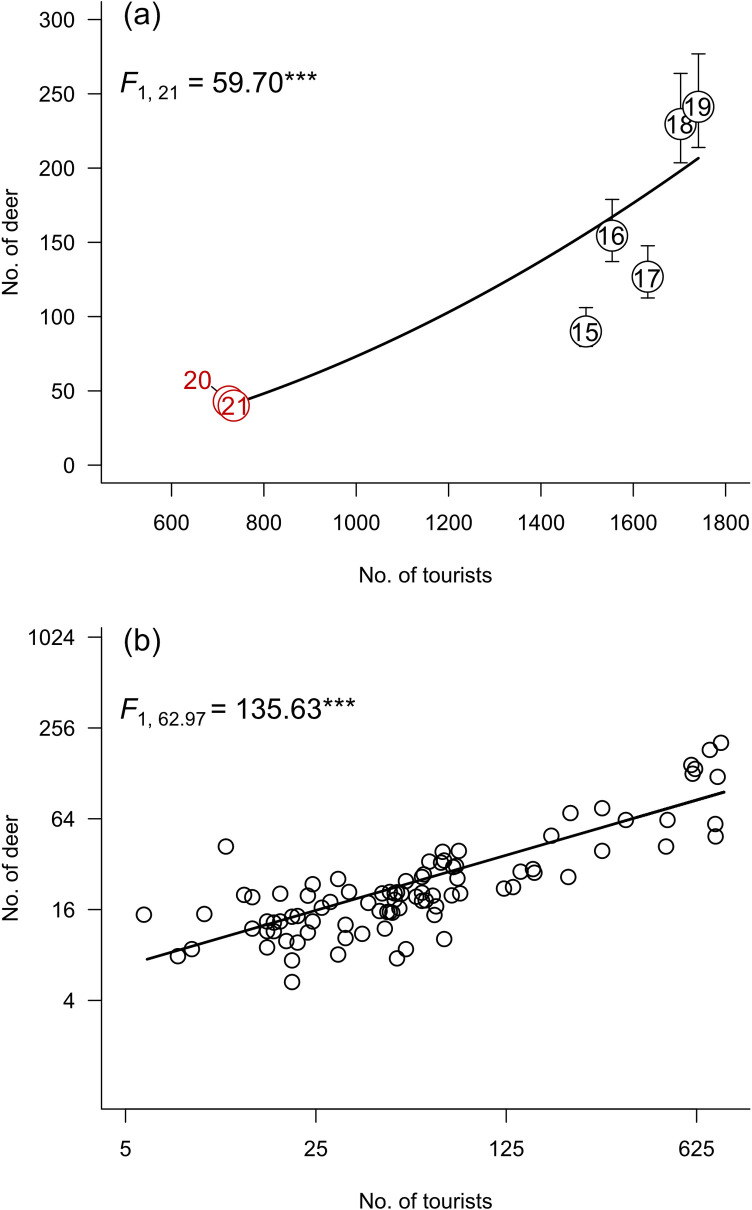
Relationship between number of deer and tourists. (a) Relationship between the annual total number of tourists visiting Nara City and the number of deer at the Todai-ji site. Numbers in the figure are the last two digits of the year. The result of the generalised linear model is shown in the upper left of the panel. Red colour indicates the pandemic period. (b) Relationship between the number of tourists and the number of deer at three sites from June 2020 to June 2021. The result of the generalised linear mixed model is shown in the upper left of the panel. Note the logarithmic scaling of both axes in Panel b. ***, *P* < 0.001.

The number of tourists visiting Nara City increased from 2015 to 2019 but decreased in 2020 and 2021 (Fig 2A). The number of deer at the Todai-ji site also increased from 2015 to 2019. An average of 167 deer used the site per survey in 2019, but the number suddenly decreased to 65 in 2020 (39% compared with the previous year) after the spread of COVID-19 (Fig 2A). Thus, the number of deer was significantly different between the months (*F*_1, 21_ = 16.60, *P* < 0.001; April (least-squares means (lower and upper SE) = 66.35 (61.04, 72.12)) < June 161.69 (136.37, 191.71)), but even considering this, the number of tourists positively affected the number of deer using the study site (*F*_1, 21_ = 59.70, *P* < 0.001; *b* ± SE = 1.87 ± 0.24; compared to *ß* = 1, *t*_21_ = 7.73, *P* < 0.001; Fig 2A). In addition, the numbers of deer at the three study sites (Todai-ji, Museum, and Ukigumo) observed monthly from June 2020 to June 2021 also positively corresponded to that of tourists (*F*_1, 62.97_ = 135.63, *P* < 0.001; *b* ± SE = 0.52 ± 0.04; compared to *ß* = 1, *t*_12.97_ = 11.65, *P* < 0.001; Fig 2B).

### Numbers of deer bows and display of aggressive behaviour

**Fig 3:**
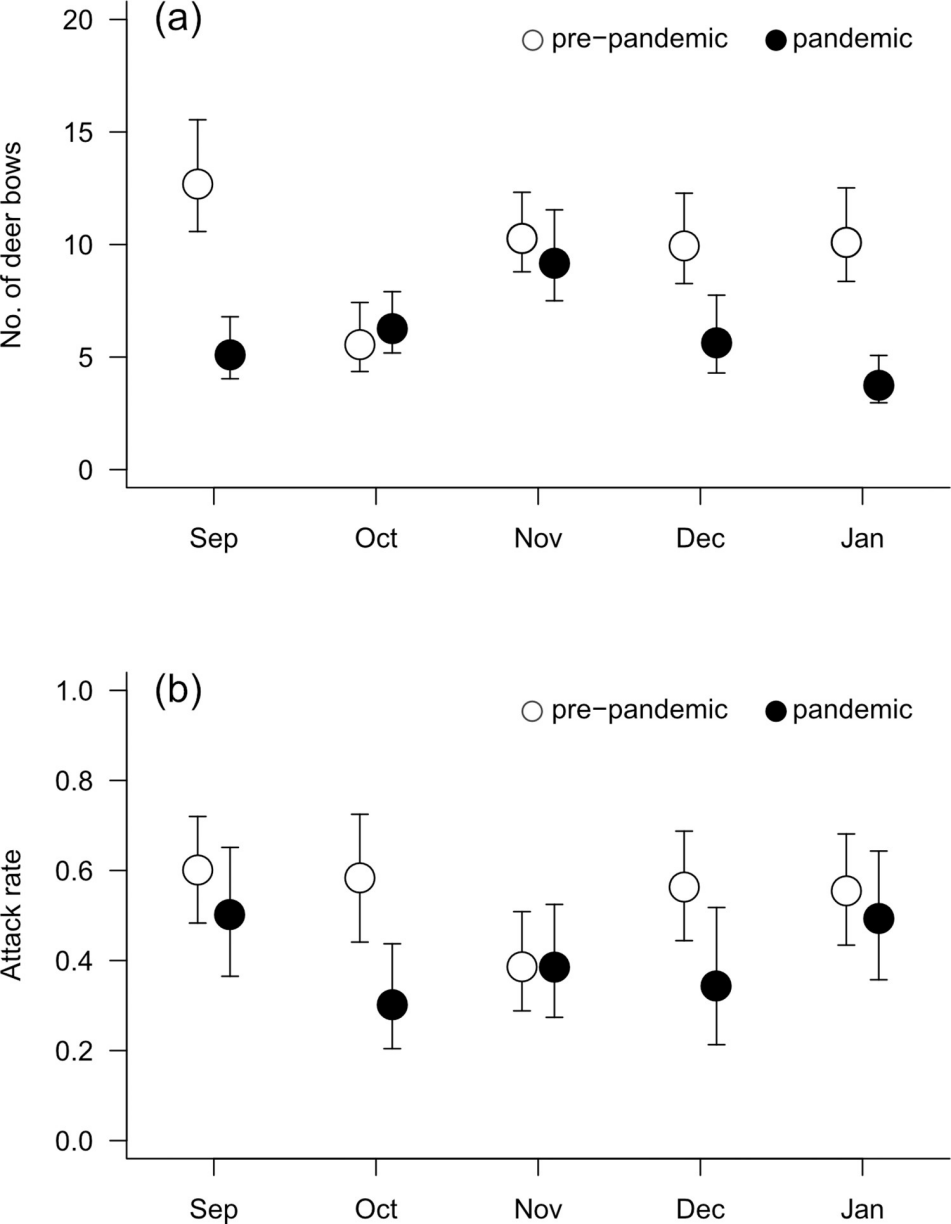
Number of deer bows and proportion of deer showing aggressive behaviour in 2016–2017 and 2020–2021. (a) Number of deer bows and (b) proportion of deer showing aggressive behaviour towards human feeders in 2016–2017 and 2020–2021 at the Museum site. All values are shown as the least-squares mean (± SE) for the month.

The number of deer bows per feeding event at the Museum site averaged 10.2 per deer in 2016–2017, and it decreased to 6.4 (62%) in 2020–2021 (Fig 3A) without any effects of month (S1 Table: year: *F*_1, 218_ = 10.93, *P* < 0.01, month: *F*_4, 218_ = 1.71, *P* = 0.15, year x month: *F*_4, 218_ = 2.18, *P* = 0.07). The attack rate (mean ± SE = 0.45 ± 0.03) was influenced by neither year nor month (S2 Table: year: *F*_1, 218_ = 3.59, *P* = 0.06, month: *F*_4, 218_ = 0.82, *P* = 0.51, year x month: *F*_4, 218_ = 0.57, *P* = 0.69; Fig 3B).

**Fig 4:**
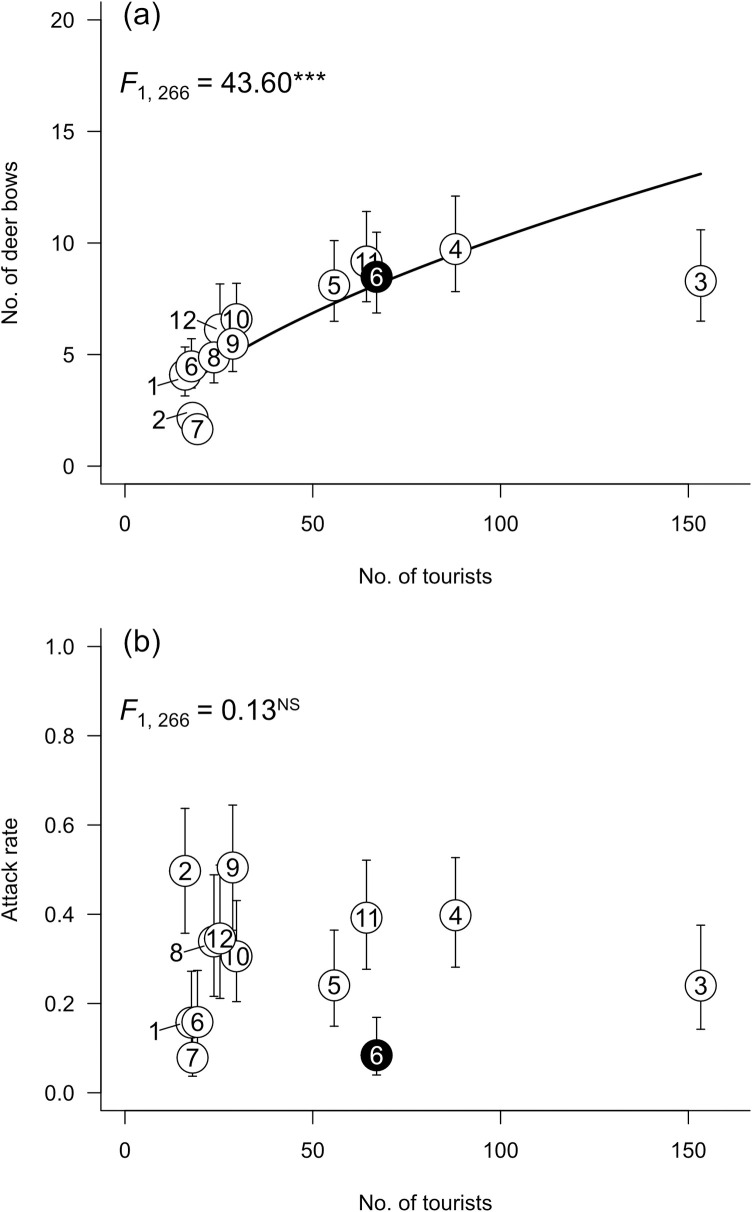
Relationships between the number of tourists, number of deer bows, and proportion of aggressive deer. (a) Relationships between the number of tourists and the number of deer bows and (b) between the number of tourists and the proportion of deer showing aggressive behaviour towards human feeders from June 2020 to June 2021 at the Museum site. Numbers in figures indicate the month. White circle with “6” indicates June 2020 and black circle June 2021. The result of the generalised linear mixed model is shown in the upper left of the panel. ***, *P* < 0.001; ^NS^, *P* > 0.05.

The number of deer bows per feeding event increased with the number of tourists at the Museum site even in the pandemic period from June 2020 to June 2021 (*b* ± SE = 0.58 ± 0.09; compared to *ß* = 1, *t*_266_ = 6.61, *P* < 0.001; Fig 4A). However, the proportion of deer showing aggressive behaviour did not depend on the number of tourists (Fig 4B). Although the feeders wore masks during the pandemic, the presence or absence of masks did not affect the behaviour of deer (S1 File).

## Discussion

The number of deer using the Todai-ji site as well as the number of tourists visiting Nara City increased from 2015 to 2019, and then both decreased in 2020–2021 (Fig 2A). As the Todai-ji site includes the gate area leading to the Great Buddha Statue, many tourists visiting Nara pass through there. Thus, it is likely that the change in the number of deer at the Todai-ji site reflected changes in the number of tourists at the Todai-ji site. The sudden decrease in the number of tourists was due to the impact of the novel coronavirus SARS-CoV-2. In Nara Prefecture, an emergency statement was declared from 16 April to 14 May 2020, and the number of tourists greatly decreased. Due to this large and unexpected decrease in the number of tourists, the opportunity for deer to obtain deer cookies also decreased. Thus, the novel coronavirus likely affected the number of deer using the Todai-ji site through the restriction of human activities.

Even during the pandemic period from June 2020 to June 2021, the number of deer at the study sites varied with the number of tourists visiting them (Fig 2B). The number of tourists increased from June to November 2020, then decreased from December to February 2021, and increased again from March (Fig 4A). As the number of tourists fluctuated, deer changed their habitat use in a relatively short period of time. Deer in Nara Park inhabit grassland, forest, bareland, etc., and feed on short-grass vegetation (mainly *Zoysia japonica*), fallen leaves, and acorns [29, 30]. Compared with such natural food, human-derived food items, including deer cookies, comprise only a small percentage of deer feeding activity throughout the year (annual average of 2.1%) [31]. However, deer prefer deer cookies to grass and acorns because deer cookies are made from rice bran and have a high nutritional value [31–33]. Thus, the deer in Nara Park often visit human-associated areas such as our study sites to obtain cookies.

The number of deer bows per feeding event was smaller during the pandemic (2020–2021) than in the pre-pandemic (2016–2017) period (Fig 3A). In addition, even during the COVID-19 pandemic (June 2020–June 2021), the number of deer bows also increased with the increase in the number of tourists (Fig 3A). These results indicate that the number of tourists affected the deer’s bowing behaviour via opportunities to feed on deer cookies. In contrast, the proportion of deer showing aggressive behaviours did not vary with the number of tourists either annually or monthly. Although both bowing and aggressive behaviour are pointed towards human feeders, the former behaviour is perceived favourably by humans but the latter is not. Thus, aggressive behaviour is less likely to be reinforced by repeated rewards than bowing behaviour, likely causing the different responses of these behaviours to changes in the number of tourists.

This study revealed that the novel coronavirus affected both the number of sika deer that use Nara Park and their bowing behaviour. Although the data before June 2020 (i.e., before the pandemic) are limited, the results showed that deer are sensitive to changes in human activities and that they can respond quickly to such changes. Moreover, contrary to most preceding studies that reported effects of anthropause (hence effects of humans) on wild animals [11, 12, 16–18, 34], the sika deer in Nara Park decreased human-mediated habitat use and the frequency of human-related behaviour in response to the anthropause. Bowing behaviour is unique among deer in Nara Park and apparently has been developed as a means of interspecific communication between humans and deer [24]. Thus, it may be an ephemeral phenomenon that will eventually disappear when continuous interaction with humans stops, as suggested in the case of a cooperative fishery between dolphins and fishers [21]. As our study is largely observational, the effects of human interactions on the frequency of the deer bowing should be experimentally tested in future.

## Conclusion

In modern urban or human-mediated ecosystems, animals are affected by human activities and have developed acquired behaviours through interactions with humans. In such cases, the anthropause caused by the COVID-19 pandemic may have had effects on animals that rely on humans. Our study demonstrated that the pandemic affected the habitat use and behaviour of the sika deer. The deer have interacted with humans since the 13th century in Nara Park [35]. Other studies also reported that the anthropause caused by the COVID-19 pandemic affected wildlife populations [11, 17, 18, 22, 34] or their behaviours [12, 16, 17, 22]. These studies demonstrate the need to highlight such responses to fully understand the relationship between humans and wildlife. The pandemic caused by the novel coronavirus is important in considering how we should interact with wildlife in the future, not only because the coronavirus has moved from wild animals to humans [4, 36–38] but also because it affects wild animals in various ways through anthropause.

## Supporting information

S1 Table Results of the generalised linear mixed model on the effects of year (2016–2017 or 2020–2021) and month (from September to January of the following year) on the number of deer bows. (DOCX)

S2 Table Results of the generalised linear mixed model on the effects of year (2016–2017 or 2020–2021) and month (from September to January of the following year) on the proportion of deer showing aggressive behaviour towards human feeders. (DOCX)

S1 File Results of the generalised linear mixed model on the effects of mask wearing on the number of deer bows and the presence/absence of aggression display by the deer. (DOCX)

S1 Data Data underlying all main and supporting information figures. (XLSX)

S1 Video Bowing behaviour of sika deer in Nara Park. Video by Haruka Uehara.
(MP4)
