# Supplementary material for: Impacts of the novel coronavirus SARS-CoV-2 on wildlife behaviour via human activities
Source: PLoS One. 2023 May 16;18(5):e0285893. doi: 10.1371/journal.pone.0285893 (PMC10187922; doi:10.1371/journal.pone.0285893)
Supplement: S2 Table — (DOCX) [file pone.0285893.s002.docx]

**Supporting information**

**S2 Table** Results of the generalized linear mixed model on the effects of year (2016–2017 or 2020–2021) and month (from September to January of the following year) on the proportion of deer showing aggressive behaviour towards human feeders.

| Factor | df | *F* |
| --- | --- | --- |
| Year | 1, 218 | 3.59 |
| Month | 4, 218 | 0.82 |
| Year × month | 4, 218 | 0.57 |
