# Supplementary material for: Impacts of the novel coronavirus SARS-CoV-2 on wildlife behaviour via human activities
Source: PLoS One. 2023 May 16;18(5):e0285893. doi: 10.1371/journal.pone.0285893 (PMC10187922; doi:10.1371/journal.pone.0285893)
Supplement: S1 File — (DOCX) [file pone.0285893.s003.docx]

**Supporting information**

**S1 Analysis** Fifteen deer (9 females and 6 males) were studied on 4 January 2023 for the number of bows and display of aggressive behaviour towards the feeder (Haruka Uehara) with and without wearing a mask. The number of deer bows and the display of aggression were analysed with GLMMs. Analyses of the number of deer bows and display of deer aggression assessed the effects of wearing a mask as fixed factors and deer sex (female or male) and individuals as random factors. The number of bows and the presence/absence of aggression by the deer were analysed using negative binomial and binomial distributions and ln-link and logit link functions, respectively.

There were no significant effects of mask wearing in either case (deer bowing: *F*_1, 15.45_ = 0.38, *P* = 0.55, deer aggression: *F*_1, 28_ = 2.06, *P* = 0.16).
